# Impact of obesity and an obesogenic environment on cardiotoxin‐induced damage and recovery of human myotubes

**DOI:** 10.1113/EP092268

**Published:** 2026-01-06

**Authors:** Brian P. Sullivan, Lundon C. Burton, Allison Ellis, Christopher K. Kargl, Deborah Shera, Shihuan Kuang, James F. Markworth, Timothy P. Gavin

**Affiliations:** ^1^ Department of Health and Kinesiology, Max E. Wastl Human Performance Laboratory Purdue University West Lafayette Indiana USA; ^2^ Department of Medicine University of Pennsylvania Philadelphia Pennsylvania USA; ^3^ Department of Endocrinology University of Illinois Chicago Chicago Illinois USA; ^4^ Department of Sports Medicine and Nutrition University of Pittsburgh Pittsburgh Pennsylvania USA; ^5^ Department of Orthopaedic Surgery Duke University Durham North Carolina USA; ^6^ Department of Animal Sciences Purdue University West Lafayette Indiana USA

**Keywords:** muscle regeneration, myoblast, Obesity

## Abstract

Obesity (BMI ≥ 30 kg/m^2^) reduces skeletal muscle quality and impairs the myogenic response to muscle damage. The present study investigated if differences exist in myotubes from individuals with (OB) or without (LN) obesity incubated in control (bovine serum albumin [BSA]) or obesogenic (Ob) medium, at baseline or in response to cardiotoxin (CTX)‐induced damage and recovery. Differentiated, primary human myotubes from LN and OB donors were cultured in control (BSA‐DM) or (Ob‐DM) (250 µM palmitate, 250 µM oleate, 100 nM insulin, and 2.5 ng/mL tumour necrosis factor) differentiation medium (DM) for 48 h and treated with 1.0 µM CTX or vehicle control for 1 h (immediately post [IP]). Myotubes recovered in Ob (Ob‐GM) or BSA (BSA‐GM) growth medium (GM) for 3 days (3D). At baseline, myotubes (LN and OB) incubated in Ob‐DM had 5% lower fusion index (FI) and nuclei/tube than BSA‐DM. At IP post‐CTX, there were no differences in FI or membrane integrity (lactate dehydrogenase), but the reduction in cell viability was greater in OB than LN myotubes and greater in myotubes (LN and OB) incubated in Ob‐DM than BSA‐DM. At 3D post‐CTX, total nuclei, FI, and nuclei/tube were ∼15% lower in myotubes (LN and OB) incubated in Ob‐GM than BSA‐GM. Expressed as recovery (3D‐IP) post‐CTX, Ob‐GM lowered total nuclei and FI in myotubes without differences between LN and OB. In human myotubes, impaired formation and recovery following damage in obesity appear primarily due to the obesogenic environment rather than inherent, individual differences.

## Abstract (highlights)

- **What is the central question of this study?**
  Obesity is associated with reduced skeletal muscle quality and impaired skeletal muscle regeneration: are decrements in skeletal muscle quality and muscle regeneration observed in obesity due to cell origin or the obesogenic environment?
- **What is the main finding and its importance?**
  In myotubes isolated from humans with and without obesity, some obesity associated decrements can be ascribed to cell origin, such as a reduction in mitochondrial content. However, poor recovery following injury appears to be primarily attributed to the obesogenic environment.

## 1 INTRODUCTION

Skeletal muscle of persons with obesity (BMI ≥30 kg/m^2^) is associated with morphological and biochemical alterations that result in reduced skeletal muscle quality, dysregulated metabolism and impaired muscle mass maintenance (Gavin et al., 2005; Malenfant et al., 2001; Sachs et al., 2019; Sullivan et al., 2020). The maintenance of skeletal muscle mass in obesity is of vital importance to preserve muscle quality and reduce the risk of sarcopenic obesity. In individuals with obesity, lipids ectopically accumulate in non‐adipose tissues, including skeletal muscle. High levels of intermuscular adipose tissue (IMAT) are correlated with macrophage accumulation in skeletal muscle of persons with obesity, promoting reduced insulin sensitivity and chronic inflammation in skeletal muscle (Patsouris et al., 2014; Sachs et al., 2019). IMAT contributes to reductions in skeletal muscle quality in persons with obesity by directly modulating skeletal muscle contractile function (Biltz et al., 2020). Obesity is also associated with increasing intramyocellular lipids, increasing membrane rigidity, and decreasing membrane integrity (Kahn et al., 2021; Knoblauch et al., 2013).

Intercellular communication is a vital process that regulates the physiological function of neighbouring cells. IMAT has a distinct pro‐inflammatory secretome, more akin to visceral than subcutaneous adipose tissue, secreting elevated proinflammatory cytokines and lipids (Ma et al., 2013; Sachs et al., 2019). Inflammatory macrophages aggregate around IMAT and secrete a plethora of inflammatory molecules (Fink et al., 2014; Kahn et al., 2021). Together these secretomes bathe skeletal muscle in an excess of lipids and pro‐inflammatory cytokines. Lipid overload and chronic inflammation are associated with negative regulation of muscle metabolism and impairments in muscle regeneration (Akhmedov & Berdeaux, 2013). Following muscle damage the inflammatory response is dysregulated (Akhmedov & Berdeaux, 2013; Brown et al., 2015; Nguyen et al., 2011), and satellite cell proliferation is markedly reduced in obese rodent models (D'Souza et al., 2015; Fu et al., 2016; Xu et al., 2018). Reductions in satellite cell activity and the dysregulated inflammatory response in individuals with obesity may be due to chronic exposure to elevated inflammatory cytokines and lipids from accumulated macrophages and IMAT.

Currently our understanding of how obesity reduces membrane integrity and alters satellite cell function during muscle regeneration in humans is limited. Studying the mechanisms of skeletal muscle damage and repair in human subjects is challenging, and consequently the majority of in vivo human research induces only moderate amounts of muscle damage and relies on indirect markers of muscle damage (Nosaka et al., 2002). Due to the difficulties associated with safety and efficacy of in vivo human experiments, most research on muscle damage and regeneration in humans is performed in vitro.

While it appears that some donor metabolic characteristics can be retained in primary myotubes, such as impaired lipid oxidation in obesity and diabetes (Boyle et al., 2012; Kim et al., 2000), the magnitude of observed in vivo impairments are reduced in vitro, particularly for normoglycemic, obese individuals (Aas et al., 2013; Gaster, 2007; Gaster et al., 2004). However, many metabolic perturbations present in vivo can be observed in vitro by treatment with lipids, insulin or inflammatory cytokines (Boyle et al., 2012; Maples et al., 2015; Reyna et al., 2008). Consequently, we investigated three interrelated, but separate questions in primary human myotubes: (1) do inherent differences exist between individuals with or without obesity (cell origin) when incubated in control or obesogenic (Ob) (elevated fatty acids, insulin, and tumour necrosis factor [TNF]) medium; (2) is cardiotoxin (CTX)‐induced myotube damage different by cell origin or incubation medium; and (3) is myotube recovery from CTX‐induced damage different by cell origin or incubation medium? We hypothesized in response to CTX treatment: (1) myotubes from individuals with obesity would demonstrate poorer outcomes than myotubes from individuals without obesity; and (2) myotubes from individuals with and without obesity cultured in an Ob medium would demonstrate poorer outcomes than cultured in standard medium.

## 2 METHODS

This study was approved by the Purdue University Institutional Review Board in accordance with the *Declaration of Helsinki*. Written informed consent was obtained from all subjects prior to their participation in the study.

### 2.1 Isolation of primary human myoblasts

Primary myoblasts were isolated as previously described (Kargl et al., 2019). Briefly, muscle biopsies were performed on the vastus lateralis of seven fasted individuals with (OB) and seven fasted without (LN) obesity using the modified Bergstrom technique. Muscle tissue was minced and digested in dispase II (Sigma‐Aldrich, St Louis, MO, USA) and Collagenase type 2 (Worthington Biochemical Corp., Lakewood, NJ, USA) for 20–30 min at 37°C. Filtered cells were pelleted and resuspended in Skeletal Muscle Growth Media (GM; Cell Applications Inc., San Diego, CA, USA) and plated on an uncoated dish for 90 min. Unattached cells were transferred to a collagen coated plate and grown to a minimum of 60% confluence in GM. Myoblasts were purified by magnetic activated cell sorting (MACS) with CD56^+^ microbeads (Miltenyi Biotec, USA). The purified myoblasts were cultured in GM for use in the experiments or frozen in liquid nitrogen for future use. All cells used in the experiments were early passage and matched between groups.

### 2.2 Differentiation and treatment of myoblasts

For all experiments, passage 4 myoblasts were seeded on 6‐ and 24‐well plates and grown in GM until 80% confluent. Myoblasts were terminally differentiated in differentiation medium (DM) (Dulbecco's modified Eagle's medium supplemented with 2% horse serum) for 6 days. To identify the dosage of CTX, myotubes were treated with varying dosages from 0 to 1.0 µM CTX (Sigma‐Aldrich). Our pilot experiments confirmed a dose of 1.0 µM CTX induced myotube damage while a significant portion of cultured cells‐maintained viability. A dose of 1 µM of CTX for 1 h has been shown to induce a robust drop in myotube number and fusion index as well as an approximate 5‐fold increase in lactate dehydrogenase (LDH) in the medium (Langone et al., 2014).

To assess the impact of an Ob medium on myotube damage and recovery, 6 days differentiated myotubes were incubated for 48 h in Ob DM (Ob‐DM) (250 µM palmitate, 250 mM oleate, 100 nM insulin and 2.5 ng/mL TNF) or control DM, containing an equivalent amount of bovine serum albumin (BSA) and phosphate buffered saline (PBS) (BSA‐DM). At the conclusion of the 48 h incubation period, cells were treated with 1 µM CTX or an equivalent volume of vehicle (PBS) for 1 h. Cells were then washed with PBS 3 times and collected for respective assays. To assess how myotubes reform and regrow after damage, damaged myotubes were cultured for an additional 3 days in Ob GM (Ob‐GM) or control GM (BSA‐GM).

### 2.3 Preparation of Ob and control media

Ob medium consisted of 250 µM palmitate, 250 µM oleate, 100 nM insulin and 2.5 ng/mL TNF in either DM or GM. Separately, palmitate and oleate were dissolved in 150 mM NaCl and heated to 70°C or 55°C, respectively. Once dissolved, palmitate and oleate were added to a 37°C, 0.17 mM BSA solution and stirred for 1 h. The final solution was diluted to create a final stock solution of 2 mM and pH adjusted with NaOH to 7.4. Control conditions contained an equivalent amount of BSA. Sodium oleate, sodium palmitate, human insulin, human TNF, CTX and BSA were obtained from Sigma‐Aldrich.

Oleate and palmitate were conjugated to BSA as previously described (Pike Winer & Wu, 2014). The dosage of 250 µM replicates hyperlipidaemia associated with obesity with equivalent concentrations of palmitate and oleate to replicate the ratio of saturated fatty acids/unsaturated fatty acids and palmitate/oleate (1:1) regularly observed in obesity and type 2 diabetes (Maples et al., 2015; Sobczak et al., 2019; Tran et al., 2016). The final molar ratio of oleate/palmitate to BSA was 3:1 which is close to what is commonly observed in human sera (Spector, 1975).

Insulin was added to the Ob medium at 100 nM to replicate elevated levels in obesity. Chronic exposure to elevated insulin leads to impairments in insulin sensitivity, a common trait of obesity (DeFronzo & Tripathy, 2009). To replicate the chronic inflammation that accompanies obesity (Pedersen & Febbraio, 2012), we included TNF in the Ob medium at a dose of 2.5 ng/mL. This low dose of TNF does not impair differentiation as severely as higher doses (Zhao et al., 2015).

### 2.4 Mitochondrial content

Mitochondrial content was assessed fluorometrically via MitoTracker Green FM in primary human myotubes according to the manufacturer's instructions (Thermo Fisher Scientific, Waltham, MA, USA). Briefly, differentiated myotubes were incubated with 100 nM of MitoTracker Green FM staining solution for 30 min at standard culture conditions. Cells were then washed with PBS and fresh medium was added to each well before being assessed on a fluorescence plate reader (excitation: 490 nm; emission: 516 nm) (SpectraMax Plus 384, Molecular Devices, San Jose, CA, USA).

### 2.5 Lactate dehydrogenase assay

The damage induced by CTX administration was quantified via LDH assay (CyQUANT, Thermo Fisher Scientific) according to the manufacturer's instructions and as previously described (Langone et al., 2014). The LDH assay quantifies cytotoxicity by measuring the production of red formazan as part of a coupled enzymatic reaction in which LDH catalyses the conversion of lactate to pyruvate via NAD^+^ reduction and the oxidation of NADH by diaphorase and as such is an index of membrane integrity. Immediately following CTX treatment, 50 µL of medium was transferred to a 96 well plate and mixed with 50 µL of the LDH reaction mixture and incubated at room temperature for 30 min. Absorbance was measured at 680 and 490 nm. The 680 nm absorbance was subtracted from the 490 nm absorbance for each well. A fold change was calculated by setting the vehicle‐treated control myotubes to 1.

### 2.6 Cell viability

The viability of human myotubes following CTX administration was assessed via a 3‐(4,5‐dimethylthiazol‐2‐yl)‐2,5‐diphenyltetrazolium bromide (MTT) assay as previously described (Kargl et al., 2019; Nie et al., 2019). The MTT assay measures cell metabolic activity by measuring the amount of formazan formed from MTT reduction by NADPH oxidoreductases. Following CTX treatment, cells were washed with PBS and fresh medium added to each well along with 50 µL of 5 mg/mL MTT reagent and incubated at standard culture conditions (37°C, 5% CO_2_) for 2 h. The medium was decanted, and the purple formazan dye was dissolved in 100 µL of dimethyl sulfoxide per well. Absorbance was measured at 550 nm (Accuscan; Thermo Fisher Scientific).

### 2.7 Myoblast proliferation and differentiation following CTX treatment

The proliferation of undifferentiated, reserve cells was quantified by visualizing the incorporation of 5‐ethynyl‐2′‐deoxyuridine (EdU) into actively dividing DNA, 3 days following CTX administration. EdU staining was performed as previously described (Salic & Mitchison, 2008). Briefly, 2 mM EdU was added to the medium 12 h prior to completion of treatment. Following completion of treatment, cells were fixed with ice‐cold, 4% paraformaldehyde (PFA), washed and incubated in 100 mM Tris–HCl, pH 8.5 (Boston Biosciences, Royal Oak, MI, USA), 1 mM CuSO_4_ (Acros; Thermo Fisher Scientific), 2.5 mM TAMRA Azide 568 (Thermo Fisher Scientific) and 100 mM ascorbic acid (Thermo Fisher Scientific) for 30 min. Prior to imaging, nuclei were co‐stained with 4′,6‐diamidino‐2‐phenylindole (DAPI) (Thermo Fisher Scientific). Myoblasts were imaged immediately via fluorescence microscopy for co‐localization of EdU to DAPI. The percentage of proliferating cells was determined using ImageJ (National Institutes of Health, Bethesda, MD, USA). Proliferation of undifferentiated reserve cells was also quantified by calculating the change in total nuclei at 3 days post (3D) compared to the total number of nuclei immediately post (IP) CTX and expressed as a percentage.

To quantify myotube formation, staining for myosin heavy chain (MHC) and DAPI was performed. Briefly, cells were fixed in 4% PFA for 15 min and then permeabilized with 100 mM glycine for 5 min at room temperature. Cells were then incubated in blocking buffer (PBS containing 5% goat serum, 2% BSA, 0.2% triton X‐100, and 0.1% sodium azide; Cell Signaling Technology, Danvers, MA, USA) for 1 h. Myotubes were subsequently incubated with MF20 antibody (DSHB), diluted 1:30 in blocking buffer overnight at 4°C. Cells were washed with PBS and incubated with secondary antibody (AlexaFlour 568, goat anti‐mouse, IgG2B, Thermo Fisher Scientific) and DAPI at a 1:500 and 1:1000 dilution, respectively. Myotubes were visualized by fluorescence microscopy and analysed in ImageJ by blinded investigators. The fusion index (FI) is the total number of nuclei incorporated into multinucleated, MHC^+^ myotubes divided by the total number of nuclei in the field of view expressed as a percentage. The average number of nuclei per multinucleated, MHC^+^ myotube was recorded and presented as nuclei/tube. Myotube damage was quantified by calculating the change in myotube FI and nuclei/tube IP CTX compared to each subject's vehicle control for that condition expressed as a percentage. Myotube reformation was quantified by calculating the change (3D‐IP) in myotube FI and nuclei/tube at 3D CTX as a percentage of each subject's IP CTX.

### 2.8 RNA isolation, reverse transcription and qRT‐PCR

Total RNA was extracted using a Trizol reagent (Thermo Fisher Scientific) as previously described (Nie et al., 2016). For mRNA reverse transcription, first‐strand cDNA was generated by random hexamer primers with MMLV Reverse Transcriptase (Thermo Fisher Scientific). Real‐time PCR detection was performed using SYBR green based chemistry on a CFX Connect (Bio‐Rad Laboratories, Hercules, CA, USA). Primers for mRNA are listed in Table 1. Gene expression was determined with the $2^{-\Delta \Delta C_{t}}$ relative quantification method and normalized to *18S* rRNA. Housekeeping genes were validated to ensure their expression was not influenced by the experimental procedure.

**TABLE 1:** Primers used for qRT‐PCR from NCBI nucleotide.

Gene name	Gene ID	Forward (5′–3′)	Reverse (5′–3′)
*18S*	106632259	GGCCCTGTAATTGGAATGAGTC	CCAAGATCCAACTACGAGCTT
*SOD2*	6648	GCTCCGGTTTTGGGGTATCTG	GCGTTGATGTGAGGTTCCAG
*TFAM*	7019	ATGGCGTTTCTCCGAAGCAT	TCCGCCCTATAAGCATCTTGA
*Glut1*	6513	GGCCAAGAGTGTGCTAAAGAA	ACAGCGTTGATGCCAGACAG
*Glut4*	6517	TGGGCGGCATGATTTCCTC	GCCAGGACATTGTTGACCAG
*PPARα*	5465	ATGGTGGACACGGAAAGCC	CGATGGATTGCGAAATCTCTTGG
*MyoD*	4654	CGCCATCCGCTATATCGAGG	CTGTAGTCCATCATGCCGTCG
*MRF4*	4618	GGAGCGCCATCAGCTATATTG	ATCCGCACCCTCAAGATTTTC
*Myf5*	4617	CTGCCAGTTCTCACCTTCTGA	AACTCGTCCCCAAATTCACCC
*PCNA*	5111	CCTGCTGGGATATTAGCTCCA	CAGCGGTAGGTGTCGAAGC
*IGF‐1*	3479	GCTCTTCAGTTCGTGTGTGGA	GCCTCCTTAGATCACAGCTCC
*Myostatin*	2660	TCCTCAGTAAACTTCGTCTGGA	CTGCTGTCATCCCTCTGGA
*RELA*	5970	TGAACCGAAACTCTGGCAGCTG	CATCAGCTTGCGAAAAGGAGCC
*IL‐8*	3576	TTTTGCCAAGGAGTGCTAAAGA	AACCCTCTGCACCCAGTTTTC

### 2.9 Statistical analysis

A one‐way analysis of variance (ANOVA) was used to test differences in response to varying dosages (0.1, 0.5 and 1.0 µM) of CTX versus control. A two‐way, mixed‐plot factorial measures ANOVA (Cell Origin [LN and OB] vs. Ob media [BSA and Ob]) was used to analyse differences between groups. Following a significant *F*‐ratio, Fisher's LSD *post hoc* analysis was performed. Subject characteristics were analysed with Student's *t*‐test. Data reported reflect a single value for each subject. Significance was established at *P* ≤ 0.05 level and data reported as means ± SD. All data were analysed in GraphPad Prism (version 10.6; GraphPad Software, Boston, MA, USA).

## 3 RESULTS

### 3.1 Subject characteristics

Demographic characteristics are in Table 2. As designed, OB had a significantly greater BMI than LN. OB had higher fasting insulin and higher homeostasis model assessment for insulin resistance (HOMA‐IR). There was no difference in blood glucose, total cholesterol, high density lipoprotein, low density lipoprotein or triglycerides between LN and OB.

**TABLE 2:** Subject characteristics with (OB) and without (LN) obesity.

Characteristics	LN (*n* = 7)	OB (*n* = 7)
Age (year)	24.4 ± 5.3	27.1 ± 4.8
BMI (kg/m^2^)^*^	22.5 ± 2.6	40.6 ± 5.3
Glucose (mg/dL)	93.7 ± 8.2	92.1 ± 10.3
Insulin (µU/mL)^*^	8.1 ± 1.6	33.1 ± 19.3
HOMA‐IR^*^	1.9 ± 0.3	7.3 ± 4.2
Total cholesterol (mg/dL)	165.6 ± 31.0	188.4 ± 38.9
HDL (mg/dL)	44.4 ± 6.9	45.0 ± 12.7
LDL (mg/dL)	101.3 ± 29.6	114.6 ± 40.2
Triglycerides (mg/dL)	100.0 ± 39.2	144.9 ± 58.2

Data are shown as means ± SD. ^*^Significantly different. Abbreviations: BMI, body mass index; HDL, high density lipoprotein; HOMA‐IR, homeostasis model assessment for insulin resistance; LDL, low density lipoprotein.

### 3.2 Dose of CTX

**FIGURE 1:**
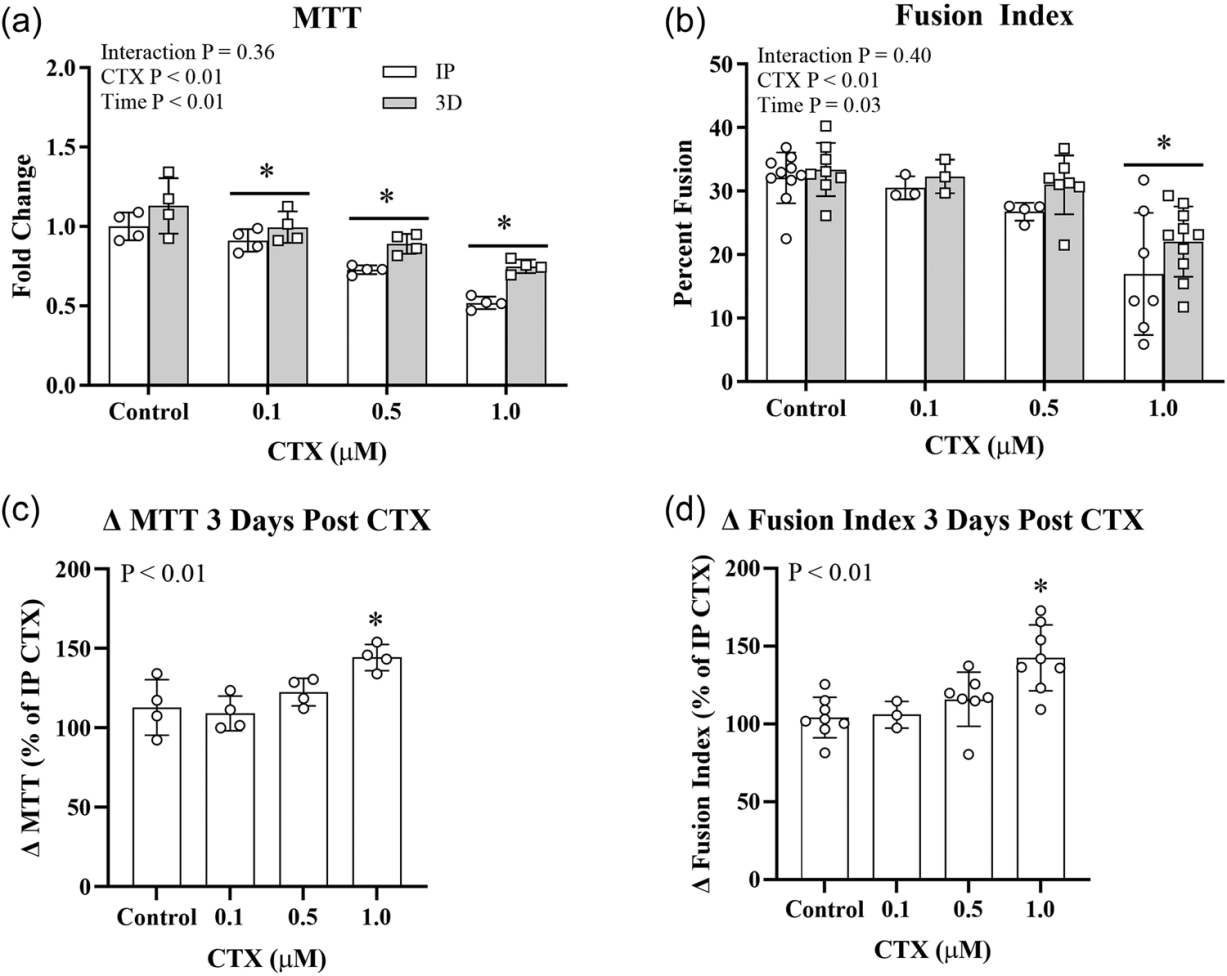
Cardiotoxin (CTX) reduces cell viability and myotube fusion. Differentiated primary human myotubes from lean donors were exposed to CTX at various doses for 1 h and either harvested immediately post‐CTX (IP) or placed in skeletal muscle growth medium (GM) for 3 days (3D) post‐CTX to facilitate myotube recovery. (a, b) Cell viability measured via 3‐(4,5‐dimethylthiazol‐2‐yl)‐2,5‐diphenyltetrazolium bromide (MTT) assay (a) and myotube fusion index (b) immediately and 3 days after exposure to various doses of CTX for 1 h. White bars, immediately post (IP) CTX; grey bars, 3 days post CTX. (c, d) The change in cell viability (c) and myotube fusion index (d) after 3 days of recovery in skeletal muscle GM compared to immediately following CTX administration. Bars show means ± SD. *n* = 3–8/group. *Significantly different from control (*P* ≤ 0.05).

The dose of CTX administered to differentiated myotubes was selected by assessing metabolic activity via MTT and myotube damage via FI following treatment with various doses of CTX (Figure 1). Compared to Control, MTT was decreased at 0.1, 0.5 and 1.0 µM CTX, while FI was decreased at 1.0 µM CTX. Both MTT and FI were increased from IP to 3D CTX at 1.0 µM. Consequently, a dose of 1.0 µM CTX was chosen for all subsequent experiments.

### 3.3 Impact of cell origin and incubation medium at baseline

**FIGURE 2:**
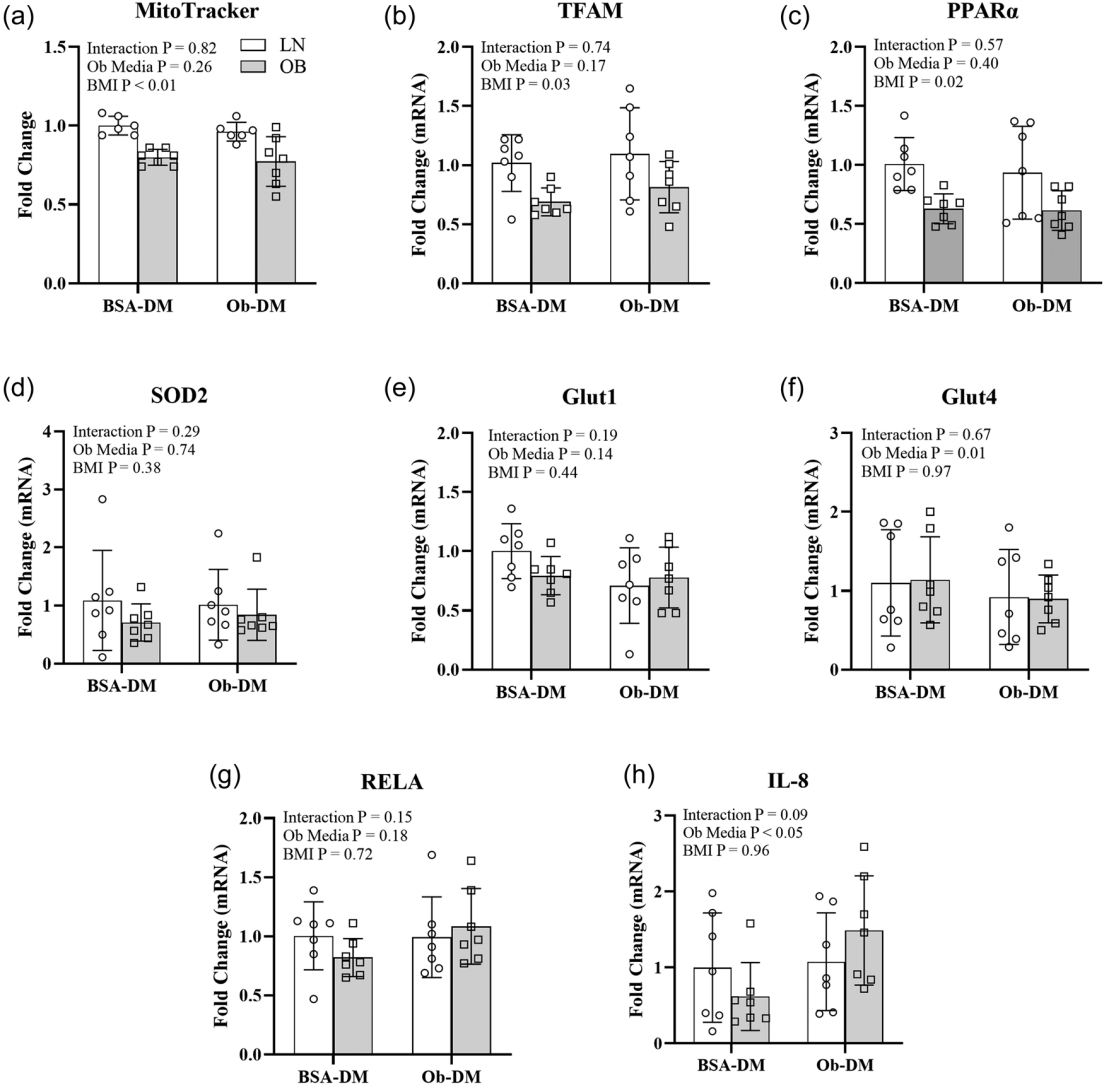
Impact of cell origin (with [OB] and without [LN] obesity) and incubation medium (control bovine serum albumin differentiation medium [BSA‐DM] and obesogenic differentiation medium [Ob‐DM]) on muscle mitochondria and gene expression. In OB, mitochondria (a) and *TFAM* (b), *PPARα* (c), and Glut1 (e) mRNA were lower compared to LN. Incubation of cells in Ob‐DM decreased *Glut1* (e) and *Glut4* (f) mRNA. There were no differences in *SOD2*, *RELA*, or *IL‐8* mRNA. Mitochondrial content was assessed fluorometrically via MitoTracker Green FM. Data were collected before treatment with CTX. White bars, LN; grey bars, OB. Bars show means ± SD. *n* = 7/group.

Myotubes from OB compared to LN exhibited 20% lower mitochondrial content and 30% lower expression of the mitochondrial regulatory genes *TFAM* and *PPARα* independent of incubation medium (Figure 2). Incubation of myotubes in Ob‐DM compared to BSA‐DM decreased gene expression of *Glut4* by 20% and increased gene expression of *IL‐8* by 60% independent of cell origin (LN and OB). There was no difference in *Glut1*, *SOD2* or *RELA* mRNA between LN and OB or BSA‐DM and Ob‐DM.

**FIGURE 3:**
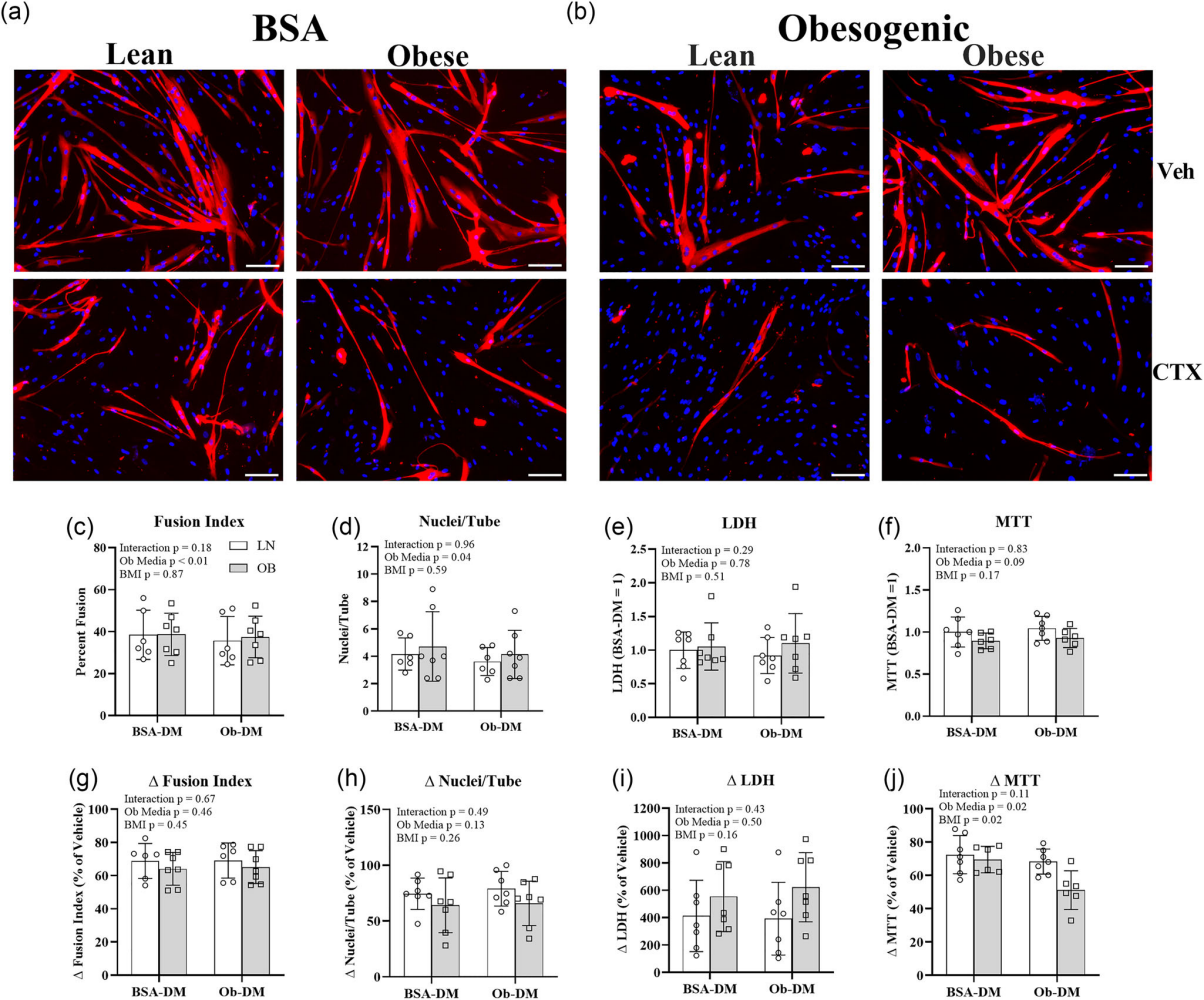
Impact of cell origin (with [OB] and without [LN] obesity) and incubation medium (control bovine serum albumin differentiation medium [BSA‐DM] and obesogenic differentiation medium [Ob‐DM]) on myotube formation and acute cardiotoxin (CTX)‐induced myotube damage. (a, b) Representative images captured immediately post (IP) of 1 h of CTX or vehicle (Veh) treatment after staining for myosin heavy chain (MHC) (red) and 4′,6‐diamidino‐2‐phenylindole (DAPI) (blue). (c, d) In Veh, incubation in Ob‐DM lowered fusion index (c) and nuclei/tube (d) compared to BSA‐DM. There was no impact of cell origin or media on lactate dehydrogenase (LDH) (e) or 3‐(4,5‐dimethylthiazol‐2‐yl)‐2,5‐diphenyltetrazolium bromide (MTT) (f). In response to CTX, the loss in MTT (j) was more in OB than LN myotubes and Ob‐DM than BSA‐DM. There was no difference in the decrease in fusion index (g), decrease in nuclei/tube (h), or increase in LDH (i) between LN and OB myotubes or Ob‐DM and BSA‐DM. OB than LN myotubes. White bars, LN; grey bars, OB. Bars show means ± SD. Scale bar = 200 µm. *n* = 6–7/group.

Before CTX treatment, incubation in Ob‐DM compared to BSA‐DM lowered myotube FI by 5% and nuclei/tube by 15% (Figure 3). There was no effect of cell origin (LN and OB) on myotube FI or nuclei/tube. There was no impact of cell origin or incubation medium on LDH or MTT.

### 3.4 Impact of cell origin and incubation medium immediately following CTX treatment

At IP CTX, there was no impact of either cell origin (LN and OB) or incubation medium (BSA‐DM and Ob‐DM) on the reduction in FI, reduction in nuclei/tube, or increase in LDH. The loss in MTT was 10% greater in OB than LN myotubes and 11% greater in Ob‐DM than BSA‐DM in LN and OB myotubes.

### 3.5 Impact of cell origin and incubation media on recovery from CTX‐induced damage

**FIGURE 4:**
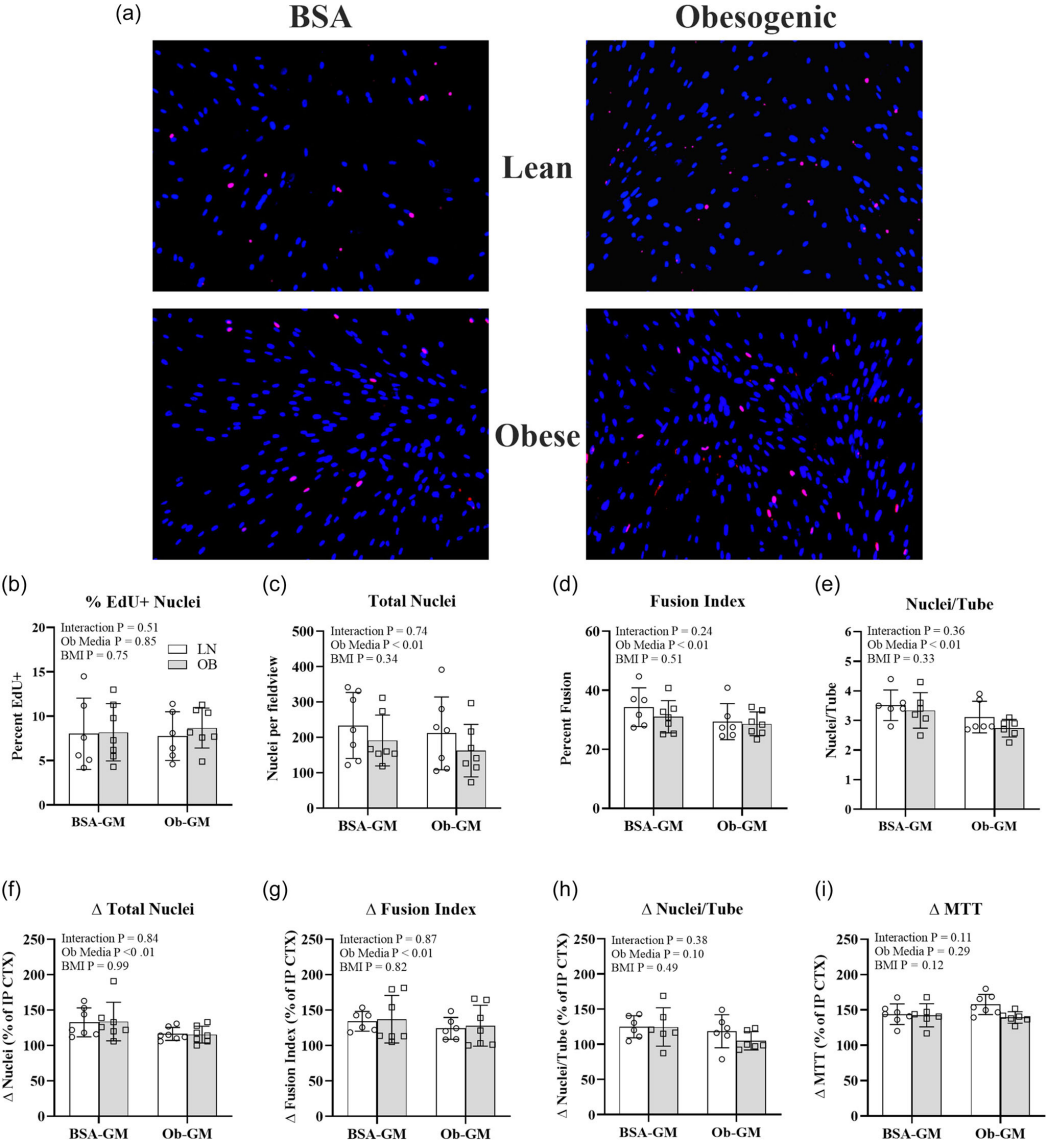
Impact of cell origin (with [OB] and without [LN] obesity) and incubation medium (control bovine serum albumin growth medium [BSA‐GM] and obesogenic growth medium [Ob‐GM]) on myotube recovery 3 days following cardiotoxin (CTX) treatment. (a) Representative images were captured at 3 days post‐CTX after staining for 5‐ethynyl‐2′‐deoxyuridine (EdU) (red) and 4′,6‐diamidino‐2‐phenylindole (DAPI) (blue). (c–e) At 3 days post‐CTX, total nuclei (c), fusion index (d), and nuclei/tube (e) were lower in Ob‐GM than BSA‐GM. (f–h) The gain in total nuclei (f), fusion index (g), and nuclei/tube (h) were lower in Ob‐GM than BSA‐GM. There was no impact of cell origin or media on the percentage of EdU+ nuclei (b) or recovery in MTT (i). White bars, LN; grey bars, OB. Bars show means ± SD. *n* = 6–7/group.

At 3D post‐CTX, incubation of LN and OB myotubes in Ob‐GM resulted in 11% fewer Total nuclei, 11% lower FI, and 15% lower nuclei/tube compared to BSA‐GM (Figure 4). However, there were no differences in the percentage of EdU^+^ nuclei due to cell origin (LN and OB) or medium (BSA‐GM and Ob‐GM) at 3D post‐CTX. Cell origin did not impact any variable at 3D post‐CTX.

Investigating recovery as the difference between 3D and IP CTX, incubation in Ob‐GM resulted in 17% fewer total nuclei and 10% lower FI compared to BSA‐GM in LN and OB myotubes. There were no differences in nuclei/tube or MTT during recovery between medium (BSA‐GM and Ob‐GM) conditions. There was no effect of cell origin (LN and OB) on any measure of recovery.

**FIGURE 5:**
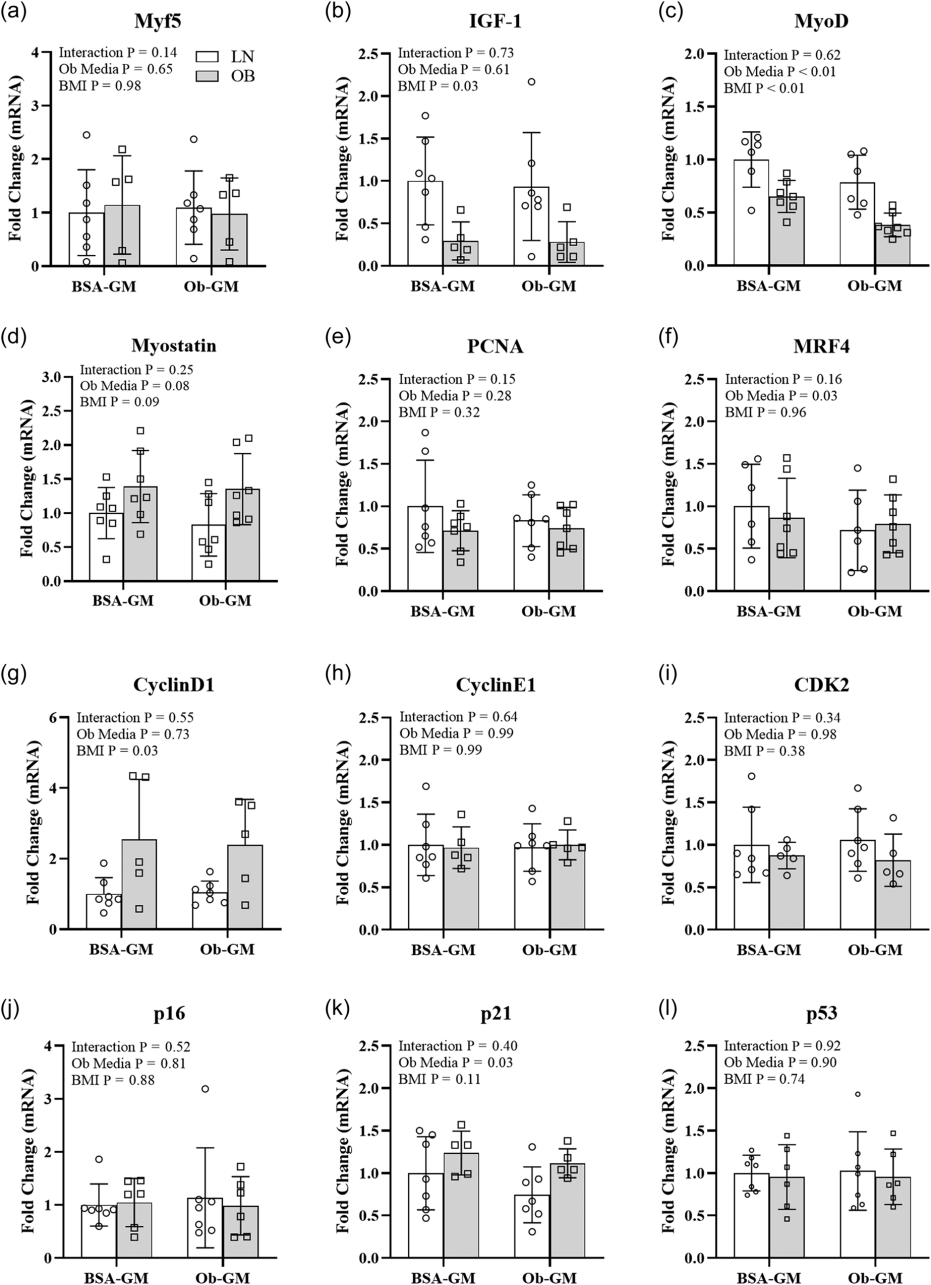
Impact of cell origin (with [OB] and without [LN] obesity) and incubation medium (control bovine serum albumin growth medium [BSA‐GM] and obesogenic growth medium [Ob‐GM]) on myotube genes associated with myogenesis and cell cycling at 3 days following cardiotoxin (CTX) treatment. Expression of *IGF‐1* (b) and *MyoD* (c) mRNA were lower and *CyclinD1* (g) was higher in OB than LN myotubes. Expression of *MyoD*, *MRF4*, and *p21* (k) were lower in Ob‐GM than BSA‐GM. There was no impact of cell origin or media on *Myf5* (a), *Myostatin* (d), *PCNA* (e), *CyclinE1* (h), *CDK2* (i), *p16* (j), or *p53* (l) mRNA. White bars, LN; grey bars, OB. Bars show means ± SD. *n* = 5–7/group.

Compared to BSA‐GM, Ob‐GM reduced the gene expression of *MyoD* by 30%, *MRF4* by 20% and *p21* by 18% at 3D post‐CTX in LN and OB myotubes (Figure 5). In OB myotubes, there was lower gene expression of *IGF‐1* by 70% and *MyoD* by 40% and greater *CyclinD1* by 40% compared to LN. There were no differences in *Myf5*, *PCNA*, *CyclinE1*, *CDK2*, *p16* or *p53* mRNA between OB and LN myotubes or BSA‐GM and Ob‐GM media.

## 4 DISCUSSION

The unique principal findings of the present study in primary human myotubes are: (1) incubation in an Ob environment reduces myotube development; (2) CTX‐induced loss of cell viability is greater in myotubes incubated in an Ob medium and in myotubes isolated from individuals with compared to without obesity; and (3) incubation in an Ob medium impairs recovery from CTX‐induced myotube damage with fewer total nuclei and lower myotube development. To our knowledge this is the first report demonstrating the impact of an Ob environment on the formation of myotubes and recovery from damage in primary human myotubes from individuals with and without obesity.

In the current report, an in vitro skeletal muscle model was employed to allow for the evaluation of unique individual characteristics retained in cell culture and the impact of an Ob environment on primary human cells. The model provided the ability to distinguish if skeletal muscle cells from individuals with obesity demonstrate myotube differences and/or whether exposing myotubes to an Ob environment might lead to detrimental changes that are not otherwise observable in cells from individuals with obesity. In addition, it provided the methodology to evaluate if exposure to an Ob environment would have detrimental consequences in cells from individuals without obesity highlighting the potential negative consequences of the Ob environment.

It has been proposed that skeletal muscle may have an ‘epi’‐memory which may be evident in muscle‐derived stem cells and thus be an appropriate model to test outcomes not possible in humans in vivo (Sharples et al., 2016). Skeletal muscle mitochondrial content in vivo can be lower in individuals with obesity compared to those without (Holloway et al., 2009), though this observation is not universal (Fisher‐Wellman et al., 2014). In the current report, mitochondrial content and glucose transporter expression were lower in cells from OB than LN donors without differences in myotube formation. Basal mitochondrial respiration has been demonstrated to be lower in myotubes from OB donors without changes in the mitochondrial citrate synthase expression or Complex I–V content (Kugler et al., 2020). However, mitochondrial Complex II and Complex IV have also been reported to be lower in myotubes from OB compared to LN donors (Lovsletten et al., 2020). Though not tested here, muscle mitochondrial capacity may have an ‘epi’‐memory in vitro which could facilitate mechanistic insight into obesity related muscle dysfunction.

Two days of incubation in an Ob medium reduced baseline fusion index and the number of nuclei per myotube. In mice, high fat feeding in vivo is associated with impaired muscle satellite cell proliferation and diminished fusion capacity in vitro and reduces muscle fibre cross section area (FCSA) (Brown et al., 2015; Geiger et al., 2020). However, in human muscle FCSA is larger in individuals with obesity than without, which is attributed in part to the continuous muscle overload present with obesity (Gavin et al., 2005; Sullivan et al., 2020). The current findings suggest that the greater FCSA with obesity in vivo occurs in an environment likely opposing fibre development due to the Ob environment. Understanding how muscle overload can promote muscle gain in an inflammatory environment could lead to improvements in the treatment of sarcopenia and cancer cachexia.

The current report investigated the impact of an Ob environment in vitro on myotube damage and recovery from CTX‐induced injury. Immediately after the 1 h CTX exposure, there were no significant differences in the loss of myotubes (FI) or membrane integrity (LDH) immediately following CTX treatment. There were greater reductions in cell viability (MTT) both by cell origin (OB > LN) and exposure to an Ob environment (Ob‐DM > BSA‐DM) suggesting that inherent as well as environmental aspects of the Ob condition may impact cellular activity. The MTT assay is dependent upon NAD(P)H‐dependent oxidoreductase enzymes that act as reducing agents to provide electrons and hydrogen ions for catabolic reactions. It is possible that greater loss of cell viability following CTX treatment in the Ob condition may be due to the inflammatory nature of the Ob environment and could contribute to poor muscle recovery from damage (Alway et al., 2023).

Muscle recovery and repair is an energy intensive process; thus, greater mitochondrial content would favour the generation of more ATP. Recovery from CTX‐induced muscle damage is impaired in muscle with lower mitochondrial content (Alway et al., 2023; LaBarge et al., 2014). In animal models, inherent mitochondrial differences are maintained during muscle regeneration (Alway et al., 2023). While not measured, it might be possible that the inherently greater mitochondrial content in LN compared to OB myotubes prior to CTX exposure would also be evident following CTX. However, myotube recovery from CTX‐induced damage was similar in LN and OB myotubes suggesting limited benefit of greater mitochondria prior to injury and recovery in the current model. It is possible that mitochondrial differences observed at baseline are not maintained in myotubes post‐CTX. Alternatively, mitochondrial differences may have less impact in primary human‐derived myotubes recovering from CTX treatment.

During differentiation in vitro, not all myoblasts differentiate. The undifferentiated myoblasts, often called myogenic reserve cells, will pause during the G_0_ phase of the cell cycle (Yoshida et al., 1998). These reserve cells are Pax7^+^/MyoD^−^, are able to re‐enter the cell cycle when reintroduced to growth conditions, and produce progeny that contribute to the formation of myotubes and the maintenance of the reserve cell population (Baroffio et al., 1996; Yoshida et al., 1998). We employed a model that was specifically designed to take advantage of these myogenic reserve cells by using BSA‐GM and Ob‐GM to facilitate cell replication and differentiation into myotubes. Interestingly, the percentage of EdU^+^ nuclei was not different between BSA‐GM and Ob‐GM. This discrepancy could likely be attributed to the timing of EdU assessment. EdU was assessed 3 days following CTX, and therefore the 12 h incubation with EdU began approximately 60 h following removal of CTX. This window of time could explain the discrepancy in the current findings (Paasuke et al., 2016). This might also account for the discrepancies between myoblast proliferation and lower *IGF‐1* mRNA (LN > OB) and lower *MyoD*/*MRF4* mRNA (LN > OB; BSA‐GM > Ob‐GM). Consistent with this, total nuclei and the change in total nuclei were lower in Ob‐GM than BSA‐GM suggesting impaired cell proliferation. Finally, fewer nuclei likely lead to lower FI and nuclei/tube at 3 days and lower recovery of FI and nuclei/tube in Ob‐GM compared to BSA‐GM.

In conclusion, poor recovery following muscle damage in obesity appears more the result of the Ob environment rather than inherent, individual differences in primary muscle cells from individuals with and without obesity. Additional work is needed to elucidate the mechanisms regulating primary, human muscle cell viability, proliferation and recovery from injury in an Ob environment.

## AUTHOR CONTRIBUTIONS

Brian P. Sullivan designed experiments, collected and analyzed data, and wrote the mansuscript. Lundon C. Burton designed experiments, collected data and analyzed data, and edited/contributed to the manuscript. Timothy P. Gavin designed experiments and edited/contributed to the manuscript. Allison Ellis, Christopher K. Kargl, and Deborah Shera contributed to data collection, experimental design, and manuscript review. Shihuan Kuang and James F. Markworth contributed to experimental design, data analysis, and manuscript review. All authors have read and approved the final version of this manuscript and agree to be accountable for all aspects of the work in ensuring that questions related to the accuracy or integrity of any part of the work are appropriately investigated and resolved. All persons designated as authors qualify for authorship, and all those who qualify for authorship are listed.

## CONFLICT OF INTEREST

None.

## Data Availability

The data that support the findings of this study are available from the corresponding author upon reasonable request.
